# Genome-wide identification of *CDPKs* in jujube and expression profile analysis in response to multiple biological processes

**DOI:** 10.3389/fpls.2025.1593446

**Published:** 2025-05-27

**Authors:** Lili Li, Ye Yuan, Bingqi Shen, Chong Chen, Lei Yang, Juan Jin, Dingyu Fan, Qing Hao

**Affiliations:** Institute of Fruits and Vegetables, Xinjiang Academy of Agricultural Sciences, Key Laboratory of Genome Research and Genetic Improvement of Xinjiang Characteristic Fruits and Vegetables, Scientific Observation and Experimental Station for Pomology of Xinjiang, Ministry of Agriculture and Rural Affairs, Urumqi, Xinjiang, China

**Keywords:** CDPKs, jujube, phylogenetic analysis, expression profiles, transcription factor

## Abstract

As calcium responders, calcium dependent protein kinases (*CDPKs*) play an important role in plant growth and development and in response to biotic and abiotic stresses. However, information on *CDPKs* in jujube (*Ziziphus jujuba* Mill.) (*ZjCDPKs*) is limited. In the current study, a total of 21 *ZjCDPKs* were identified, which are located on eight chromosomes. Gene structure and conserved motif analysis showed that all *ZjCDPKs* have similar gene structures and conserved motifs, except for *ZjCDPK9* and *ZjCDPK21*. The *CDPKs* from Arabidopsis, rice, tomato, alfalfa, and jujube were divided into eight subgroups, and the members of *ZjCDPKs* were unevenly distributed across these subgroups. Colinear analysis revealed that 12 homozygous *CDPKs* were detected between jujube and *Arabidopsis*, and 14 pairs were found between jujube and tomato. Additionally, four types of *cis*-acting elements were identified in the promoters of the *ZjCDPKs*, including hormone, stress, development, and light response elements. The expression profiles of *ZjCDPKs* at different fruit growth stages, in response to phytoplasma infection, cold, and salt stresses revealed that most *ZjCDPKs* were either up- or down-regulated. Finally, varying numbers of transcription factors were observed to interact with the promoter region of *ZjCDPK4/6/7/8/10/14/16* and showed opposite expression patterns in response to cold or salt stress. The systematic analysis of *ZjCDPKs* provides important information for further functional characterization of *CDPKs* in jujube in response to multiple biological processes.

## 1 Introduction

In nature, plants have evolved sophisticated mechanisms to respond and adapt to a wide variety of biotic and abiotic stresses (Wang et al., 2020b). Calcium (Ca^2+^), as an important second messenger, plays a crucial role in signal transduction during stress adaptation and plant growth and development (Wang et al., 2023a; Tuteja and Mahajan, 2007; Wang et al., 2020b). Three calcium sensors have been identified in plants, including calmodulin (CaM)/calmodulin-like proteins (CML), calcineurin B-like protein (CBL), and calcium dependent protein kinase (*CDPK*) (Wang et al., 2023b). Among them, the signaling system composed of CBLs and their interacting protein kinases participates in the regulation of plant response to low-temperature stress through the phosphorylation induction and decoding of Ca^2+^ signals (Ma et al., 2020). CMLs can directly bind with Ca^2+^, which is involved in the responses to numerous stresses (Wang et al., 2023b). Moreover, previous research has reported the activation of *CDPKs* by Ca^2+^ to perform phosphorylation, indicating the key role of *CDPKs* as Ca^2+^ responders (Dekomah et al., 2022a).

*CDPKs* are a class of serine/threonine type protein kinases with four characteristic domains, including a variable N-terminal domain (VNTD), a catalytic Ser/thr protein kinase domain (PKD) (which can bind the ATP phosphate donor and phosphorylates the serine and threonine residues of its substrates), an autoinhibitory domain (AIR), and a carboxyl-terminal calmodulin-like domain (CLD) (which has one to four conserved EF-hand motifs for calcium-binding) (Cheng et al., 2001; Hrabak et al., 2003; Liese and Romeis, 2013). Apart from the functions of PKD and CLD, the VNTD domain typically contains N-myristoylation sites or N-myristoylation and S-palmitoylation sites, and plays an important role in subcellular location and substrate recognition. The AIR domain, also denoted as the junction domain function, acts as a pseudo substrate to maintain *CDPK* inactive in the absence of Ca^2+^ stimulation (Hrabak et al., 2003; Hamel et al., 2014). When Ca^2+^ increases, the EF-hand can bind motifs, leading to conformation changes in CLD and the subsequent activation of the *CDPK* kinase domain, which can recognize and phosphorylate downstream targets (Dekomah et al., 2022a).

Similar to CaM/CML and CBLs, *CDPKs* play an important role in the signal transduction pathway, including secondary metabolites, hormone regulation, and stress tolerance (Dekomah et al., 2022a). For example, in maize, the overexpression of *ZmCDPK7* can enhance thermotolerance by decreasing the accumulation of hydrogen peroxide (H_2_O_2_) and malondialdehyde (MDA) (Zhao et al., 2021). In tomato, virus-induced gene silencing stimulates the silencing of *ShCDPK6*, while *ShCDPK26* shows less resistance to Botrytis cinerea, cold, and drought stress (Li et al., 2022). In potato, *StCDPK21/22* and *StCDPK3* can regulate the content of MDA and proline to facilitate drought tolerance (Dekomah et al., 2022b). During peach storage, *PpCDPK7* has been reported to interact with PpRBOH, which further mediates the Ca^2+^ and reactive oxygen species (ROS) signal cascades to enhance chilling tolerance (Zhao et al., 2022). In wheat, *TaCDPK25-U* is significantly induced by drought stress and can be positively regulated by *TaDREB3* to improve drought tolerance (Linghu et al., 2023). In *Arabidopsis*, *AtCPK28* can be activated by Ca^2+^ and phosphorylated downstream NLP7 to improve cold tolerance (Ding et al., 2022). Moreover, resveratrol levels can increase following the overexpression of *VaCPK20* or *VaCPK29* in *Vitis amurensis* cells (Aleynova-Shumakova et al., 2014). *AtCPK6* acts as a positive regulator in stomatal movement or closure by ABA and methyl jasmonate, respectively, in a signaling-dependent manner (Xu et al., 2010; Ye et al., 2013). With the development of plant genome sequencing and the important biological function of *CDPKs*, the *CDPKs* members in different plant species have been widely identified, including 34 members in *Arabidopsis thaliana* (Hrabak et al., 2003), 31 in rice (Kong et al., 2013), 29 in tomato (Wang et al., 2016), 40 in maize (Kong et al., 2013), 30 in pear (Liu et al., 2022), and 17 in peach (Zhao et al., 2022). However, the identification of *CDPKs* in jujube and their biological function has not yet been reported.

Jujube (*Ziziphus jujuba* Mill.) belongs to the Rhamnaceae family and has important economic and ecological value in China (Liu et al., 2020). Jujube is widely planted in sandy alkali arid areas, which has facilitated its advanced tolerance to salt, drought, and cold tolerance (Wang et al., 2020a; Gao et al., 2021; Wang et al., 2025). With the whole genome sequencing completed in jujube, it has become an ideal fruit tree for research on abiotic stress mechanisms. Thus, in the current study, the identification, phylogenetic analysis, gene structure, and conserved motifs of *CDPKs* in jujube were conducted. The RNA-seq data was then used to analyze the expression levels of *CDPKs* in response to various biotic and abiotic stresses and fruit growth development. The results provide a theoretical basis for clarifying the biological function of *CDPKs* in jujube.

## 2 Materials and methods

### 2.1 Identification and analysis of the physicochemical properties of the *CDPKs* in jujube

To screen the *CDPKs* members in jujube, the complete genome and annotated information files of jujube were downloaded from the NCBI database (https://www.ncbi.nlm.nih.gov), and the *CDPK* Hidden Markov model (PF00069 and PF13499) was downloaded from the InterPRO database (https://www.ebi.ac.uk/interpro/). HMMER with an *E* value of 1e^-3^ parameter was employed to search for the *CDPK* protein sequences in the jujube genome database and obtain the initial members of potential *CDPKs*. Following this, 34 *CDPKs* in *Arabidopsis* were retrieved from the TAIR database (https://www.arabidopsis.org) to perform BLASTP analysis against the jujube genome. The Conserved Domain Database (CDD) with a *P*-score cutoff 0.03 (http://www.ncbi.nlm.nih.gov/cdd/) was then employed to determine the conservative domains, remove the redundant sequence (Cao et al., 2024b), and finally obtain the *CDPK* members in jujube (*ZjCDPKs*).

The online ExPASy tool (http://web.expasy.org/protparam/) was used to determine the molecular weight (kDa), isoelectric points (pI), and other physicochemical properties of the *ZjCDPKs* (Wilkins et al., 1999).

### 2.2 Chromosomal location prediction of *ZjCDPKs*

The CDS, protein sequences (**Supplementary Table 1**), and chromosome position information of the *ZjCDPKs* were obtained from the NCBI database.

### 2.3 Gene structure and conserved motif analysis

TBtools was employed to extract the CDS and related genome formation of the *ZjCDPKs* for the visualization analysis of the gene structure (Chen et al., 2020). The MEME online tool (http://meme-suite.org/) was used to analyze the conserved motifs of the *ZjCDPKs*, with a maximum motif number of 10 and an optimal motif width for the 6–50 amino acid residues (Bailey et al., 2009). The conserved motifs were then visualized in TBtools.

### 2.4 Phylogenetic analysis of the *CDPKs* in jujube and four other species

The *CDPK* protein sequences of Arabidopsis, rice, tomato, alfalfa, and jujube were retrieved from the NCBI database. MEGA 5.0 was used to construct the phylogenetic tree using the neighbor joining (NJ) method, with a bootstrap value of 1,000 (Tamura et al., 2011). The visualization of the phylogenetic tree was improved with the iTOL online tool (https://itol.embl.de).

### 2.5 Collinearity and *cis-*acting element analysis

The genome data of *Z. jujuba* Mill., *Arabidopsis thaliana*, and *Solanum lycopersicum* were used to determine duplication events and perform collinearity analysis of the *CDPKs* using MCScanX with *Ka/Ks* value (Qi et al., 2024). The results were visualized in TBtools.

For the *cis*-acting elements analysis, the 2 kb sequences upstream of each *ZjCDPK* were extracted as the promoter region and submitted to the PlantCARE database (http://bioinformatics.psb.ugent.be/webtools/plantcare/html) to search for *cis*-acting elements (Higo et al., 1998). TBtools was used to visualize the results and create the heat maps.

### 2.6 Expression profile analysis of *ZjCDPKs* during jujube fruit development and in response to biotic and abiotic stresses

To understand the biological functions of *ZjCDPKs*, the expression profiles of *ZjCDPKs* were mined from the transcriptome data in response to low-temperature and salt stresses, phytoplasma infection materials, and different jujube fruit growth stages of ‘Jinsixiaozao’ and ‘Jinkuiwang’, respectively. The transcriptome assembly process could be referred to Cao et al. (2024a, 2025) and Jiang et al. (2025). Briefly, for cold treatment, samples of *Z. jujuba* Mill. ‘Dongzao’ and its autotetraploid were treated under a cold temperature (4°C) for 0, 6 and 24 h (Gao et al., 2021). For salt stress, 400 mM NaCl was treated on sour jujube seedlings for 0, 6, 24, and 192 h (Zhu et al., 2023). For phytoplasma infection analysis, different growth stages (S1, S2, and S3) of *Z. jujuba* Mill. ‘Pozao’ (‘PZ’) and *Z. jujuba* Mill. ‘T13’ were selected for RNA-seq analysis, where PZ_D and T13_D denote samples infected by phytoplasma, respectively, and PZ_H and T13_H denote healthy samples (Wang et al., 2022). For the jujube fruit growth analysis of ‘Jinsixiaozao’ (JS) and ‘Jinkuiwang’ (JKW), fruit from nine growth stages, namely, the early stage of young fruit (F1), the middle stage of young fruit (F2), the early stage of stone formation (F3), the stone formation stage (F4), the white mature stage (F5), the late white mature stage (F6), the quarter coloring stage (F7), the half red stage (F8), and the full red stage (F9), were selected for RNA-seq analysis (Zhao et al., 2023). The expression levels of *ZjCDPKs* were presented on a heat map in TBtools.

### 2.7 Mining and expression analysis of related transcription factor of *ZjCDPKs*

The PlantRegMap database (http://plantregmap.gao-lab.org/network.php) was employed to identify the potential transcription factors (TFs) upstream of *ZjCDPK4/6/7/8/10/14/16* and their expression profiles in response to cold and salt stresses were then evaluated (Tian et al., 2020).

## 3 Results

### 3.1 Identification, physicochemical characteristics, and chromosome locations of *ZjCDPKs*

Through the systematic and genome-wide identification of *ZjCDPKs*, a total of 21 *ZjCDPK* members were screened and denoted as *ZjCDPK1*–*ZjCDPK21* based on their chromosome positions. As shown in **Table 1**, the physicochemical characteristics (e.g., amino acid length and theoretical isoelectric point (pI) values) of the *ZjCDPKs* varied. The number of amino acids ranged from 291aa (*ZjCDPK9*) to 646 aa (*ZjCDPK19*). The molecular weight (mw) ranged from 33.15 kD (*ZjCDPK9*) to 72.70 kD (*ZjCDPK19*) and the pI values ranged from 5.20 (ZjCDPK18) to 9.03 (ZjCDPK11). In addition, the *ZjCDPKs* were located on eight chromosomes (Chr). Among them, three members of the *ZjCDPKs* (*ZjCDPK1*–*ZjCDPK3*) were located on Chr1, while *ZjCDPK8– ZjCDPK10* was located on Chr4. Two and one *ZjCDPKs* were identified on Chr2/3 and Chr8/9/12, respectively. Four members of the *ZjCDPKs* were located on Chr 11, while *ZjCDPK18*–*ZjCDPK21* did not match with any Chrs.

**Table 1:** The characteristics of *CDPKs* in Chinese jujube.

Gene name	Gene ID	Size (aa)	MW (kDa)	pI	Chromosome	Start site	Termination site
*ZjCDPK1*	LOC107419468	549	61752.03	6.05	1	13142856	13147600
*ZjCDPK2*	LOC107422810	516	58401.49	5.81	1	17240003	17243858
*ZjCDPK3*	LOC107433024	547	61375.77	6.21	1	34726940	34731327
*ZjCDPK4*	LOC107410708	530	59059.96	5.74	2	5905790	5908997
*ZjCDPK5*	LOC107412614	534	60283.69	6.5	2	26793548	26798433
*ZjCDPK6*	LOC107412864	544	60753.24	5.69	3	1927705	1931577
*ZjCDPK7*	LOC107412936	536	59732.92	5.6	3	2710718	2716459
*ZjCDPK8*	LOC107415637	524	59941.14	8.08	4	5377350	5381638
*ZjCDPK9*	LOC107415638	291	33148.81	5.27	4	5390033	5393488
*ZjCDPK10*	LOC107415799	493	55600.52	5.48	4	6802773	6806523
*ZjCDPK11*	LOC107424366	578	65409	9.03	8	6141911	6147215
*ZjCDPK12*	LOC107427186	568	63548.74	5.53	9	15299792	15305830
*ZjCDPK13*	LOC107430235	531	59439.54	5.9	11	4243697	4248879
*ZjCDPK14*	LOC107430444	572	63798.54	5.5	11	9548635	9554274
*ZjCDPK15*	LOC107430482	497	55928.77	5.41	11	9776374	9782317
*ZjCDPK16*	LOC107430894	526	59401.79	5.86	11	13519246	13526811
*ZjCDPK17*	LOC107432385	530	59988.48	6.31	12	7128171	7132262
*ZjCDPK18*	LOC107433607	622	68998.17	5.2	NW_015453408.1	39769	45173
*ZjCDPK19*	LOC107433642	646	72702.43	5.33	NW_015453408.1	153660	157696
*ZjCDPK20*	LOC107435963	487	56309.16	6.96	NW_015453584.1	26584	30613
*ZjCDPK21*	LOC107409377	307	34459.65	5.23	NW_015456732.1	5	2683

### 3.2 Conserved motifs and gene structure of *ZjCDPKs*

**Figure 1:**
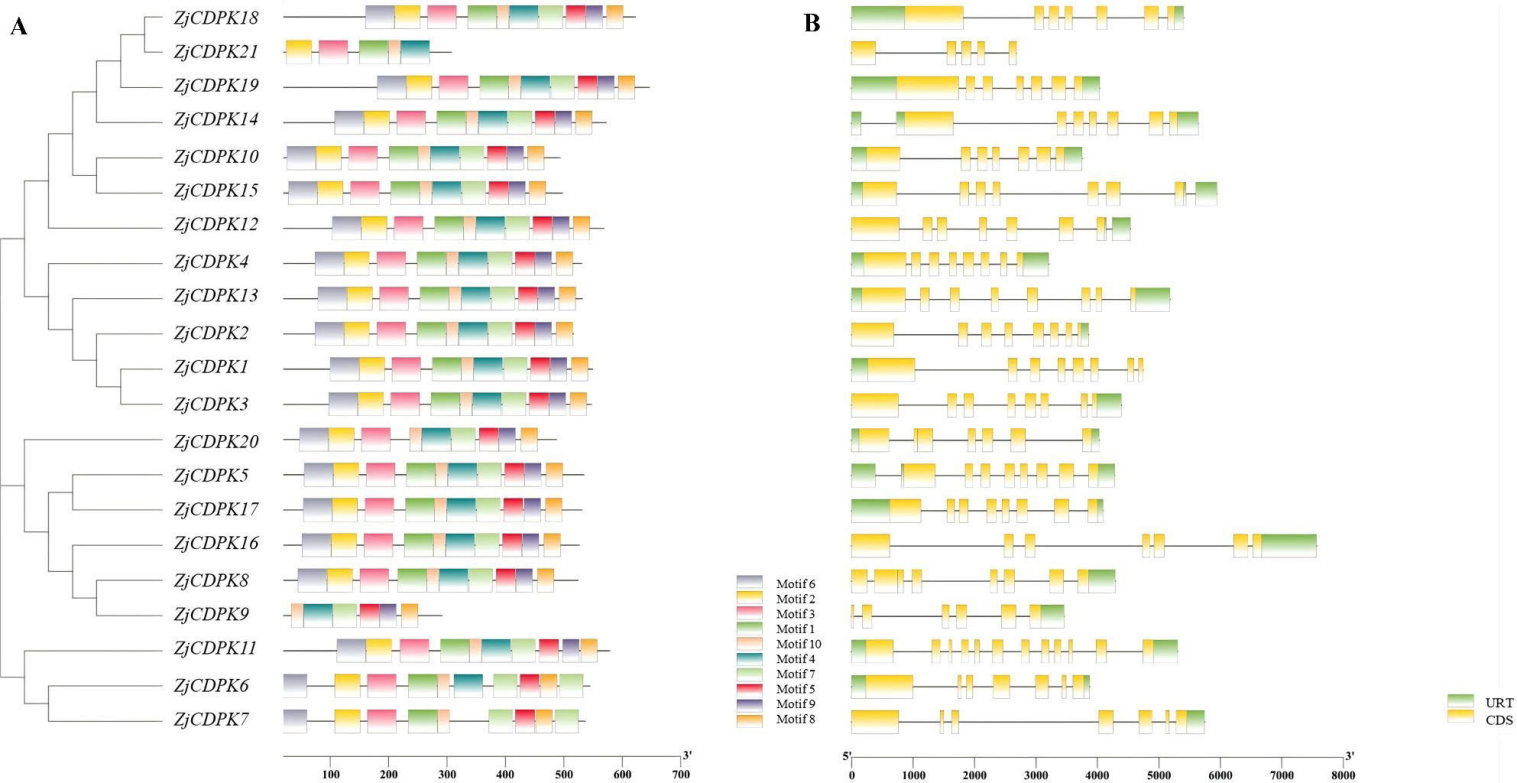
Conservative motifs and gene structures of *ZjCDPKs*. **(A)** Schematic diagram of the conserved motifs of *ZjCDPKs*. Ten motifs are represented by different colored boxes. **(B)** Gene structures of *ZjCDPKs*. Exons, introns, and UTR are represented by yellow boxes, black lines, and green boxes, respectively.

*CDPKs* have four characteristic domains, namely, VNTD, PKD, AIR, and CLD. Through MEME analysis, 10 conserved motifs were identified in the *ZjCDPKs* (**Supplementary Table 2**). As shown in **Figure 1A**, all the *ZjCDPKs* contained nine or ten conserved motifs, except for *ZjCDPK9* and *ZjCDPK21*, which only contained five and six, respectively. The upstream of motif1 was found to be the kinase domain and the auto-inhibitory junction region was observed in motif4. Moreover, the EF-hand domains were identified in the motifs. The *ZjCDPKs* exhibited a similar number and spatial distribution of motifs in the same subgroup, indicating that the *ZjCDPK* proteins have evolutionary conservatism.

Gene structure analysis showed that all *ZjCDPKs* had similar gene structures and were interrupted by 5–11 introns. In particular, nine *ZjCDPKs* had seven introns and seven *ZjCDPKs* had six introns (**Figure 1B**). *ZjCDPK9*, *ZjCDPK20*, and *ZjCDPK21* had fewer introns (five, five, and four, respectively), while *ZjCDPK11* had the largest number of introns (eleven). The similarity and diversity of the *ZjCDPKs* gene structures may indicate their similar and distinct biological functions.

### 3.3 Phylogenetic analysis of the *CDPKs*

**Figure 2:**
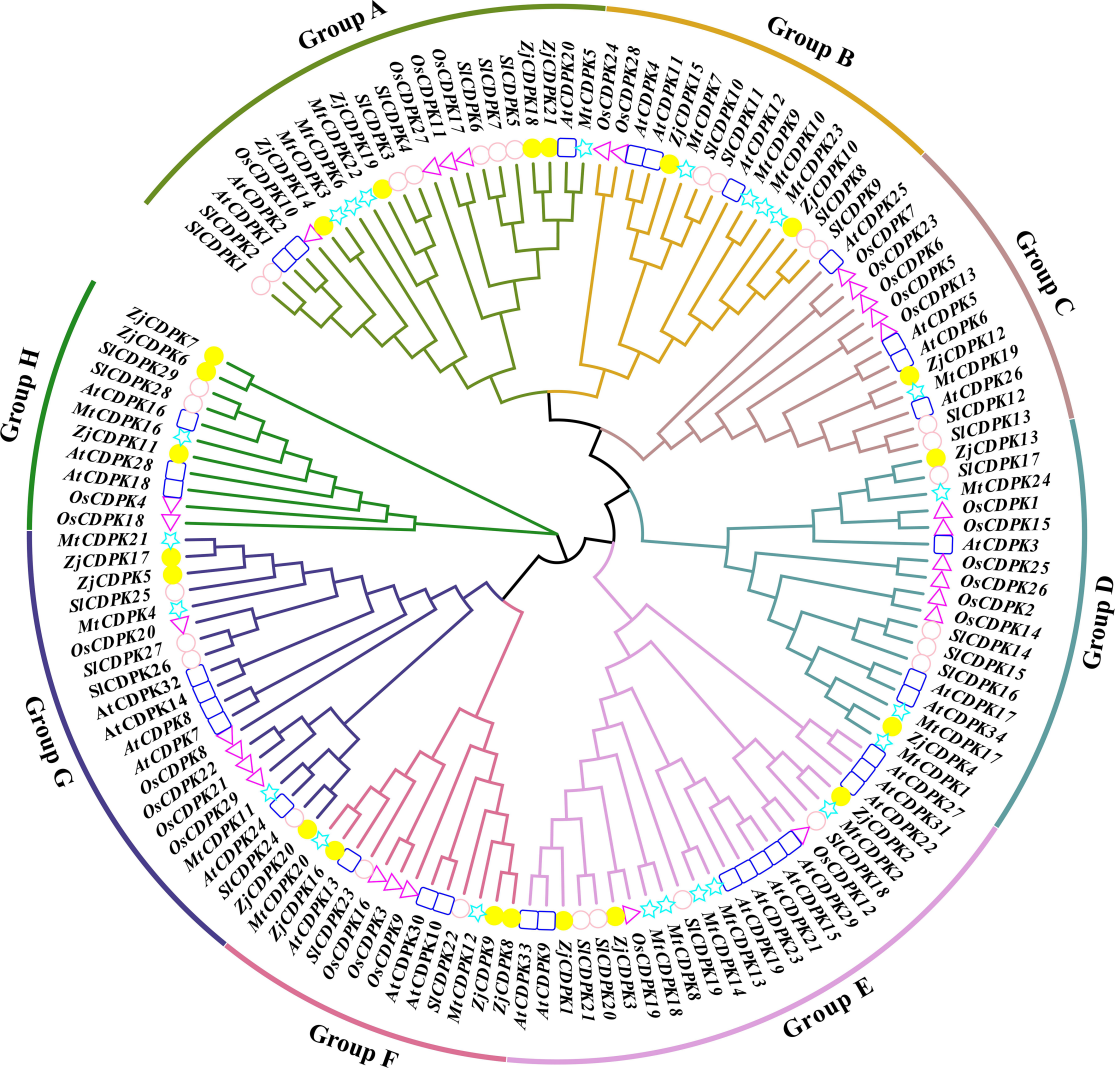
Phylogenetic tree analysis of the *CDPKs* in jujube, *Arabidopsis*, rice, tomato, and alfalfa, represented by yellow circles, blue squares, purple triangles, light purple circles, and green triangles, respectively. Different subgroups are represented by different line colors.

To study the evolutionary relationship of the *CDPKs* in jujube, *Arabidopsis*, rice, tomato, and alfalfa, we constructed an NJ phylogenetic tree using *CDPK* protein sequences from the above species. As shown in **Figure 2**, the *CDPKs* were divided into eight subgroups. The 21 *ZjCDPKs* members were distributed into different subgroups, including four in subgroup A, two in subgroup B, one in subgroup C, two in subgroup D, and three in subgroups E, F, G, and L, accounting for 18.18%, 13.33%, 8.30%, 12.50%, 12.50%, 23.07%, 15.00%, and 27.27% respectively. In *Arabidopsis*, three (13.63%), three (20.00%), three (25%), three (18.75%), ten (41.67%), three (23.07%), five (25.00%), and three (27.27%) *CDPKs* were categorized in subgroups A to H, respectively. The same was observed for rice, tomato, and alfalfa. In addition, the proportion of *ZjCDPKs* in different subgroups was similar to that of *Arabidopsis*, rice, tomato, and alfalfa, indicating that the evolution of *CDPK* in different plant species was conservative.

### 3.4 Colinear analysis of *ZjCDPKs*

**Figure 3:**
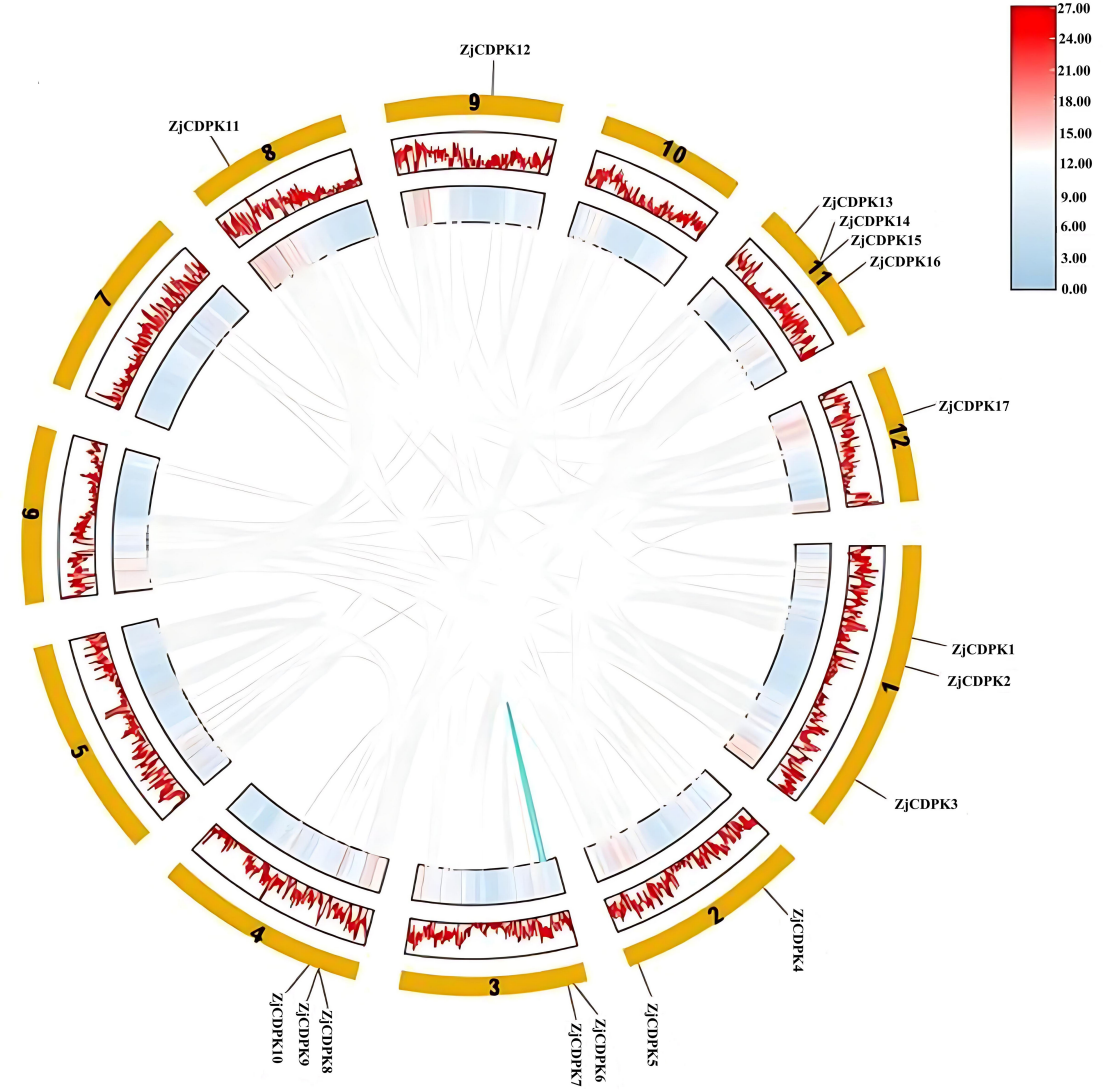
Colinear analysis of the *ZjCDPKs*. The outermost circle represents 12 chromosomes. The gray and colored connecting genes represent collinear blocks and segmental duplication events. The color lines in the middle and inner layers of the circle represent the gene density of the chromosome.

The tandem and segmental duplication functions are important in gene evolution analysis. Here, through collinearity analysis in the jujube genome, two pairs of *ZjCDPKs* (*ZjCDPK7/9* and *ZjCDPK14/15*) exhibited tandem duplication (**Figure 3**). In addition, one pair of collinearity genes (*ZjCDPK6/7*) was identified, indicating that segmental duplication occurred on the same chromosome.

**Figure 4:**
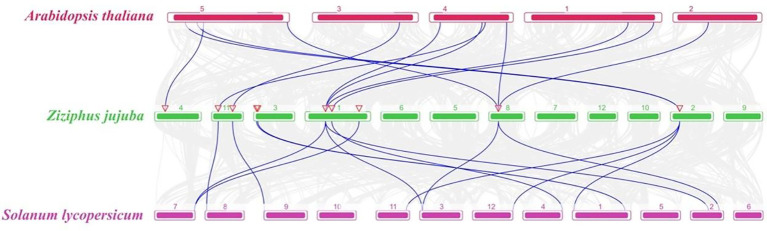
Colinear analysis of the *CDPKs* from jujube, *Arabidopsis*, and *Solanum lycopersicum*. The gray line represents the collinear blocks among jujube, *Arabidopsis*, and *Solanum lycopersicum* genomes, while the blue line represents the collinearity of *CDPK* gene pairs among these three species.

To explore the evolutionary relationship of the *CDPKs* in different species, collinearity analysis of the *CDPKs* from jujube, *Arabidopsis*, and *Solanum lycopersicum* was performed. The collinearity plot identified 12 pairs of homozygous genes between jujube and *Arabidopsis*, while two *ZjCDPK* genes (*ZjCDPK1* and *ZjCDPK11*) simultaneously formed homozygous gene pairs with three *Arabidopsis CDPKs* (**Figure 4**). In addition, a total of 14 homologous gene pairs were detected in jujube and tomato, and *ZjCDPK1* and *ZjCDPK4* also formed homologous gene pairs with three tomato *CDPKs*. This suggests that these genes may play an important role in the phylogeny of the *CDPKs*.

### 3.5 *Cis-*acting element analysis of *ZjCDPKs*

**Figure 5:**
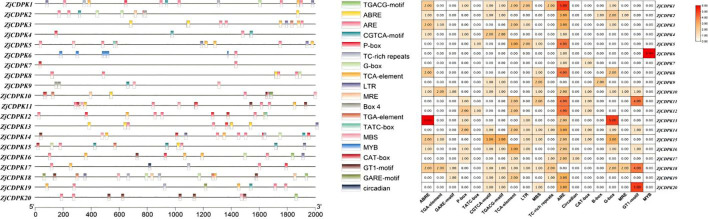
Identification of the *cis*-acting elements of the *ZjCDPK* promoters. The different types of *cis*-acting elements in the promoter region of the *ZjCDPKs* are represented by different colors. The different colors in the grid represent *cis*-acting elements.

To investigate the possible transcriptional regulation network of the *ZjCDPKs*, TBtools was used to extract the 2 kb sequences upstream of the *ZjCDPKs*. These sequences were uploaded to PlantCARE to identify the *cis*-acting elements (**Supplementary Table 3**). As shown in **Figure 5**, four types of *cis*-acting elements were identified in the promoters of *ZjCDPKs*, including hormone, stress, development, and light response elements. Hormone-related *cis*-acting elements mainly included the abscisic acid response element (ABRE), the auxin response element (TGA element), the gibberellin response element (GARE motif/P-box/TATC box), the methyl jasmonate response element (CGTCA motif/TGACG motif), and the salicylic acid response element. The methyl jasmonate response element was most abundant in the *ZjCDPKs* promoters (21 occurrences), followed by the salicylic acid response element (10 occurrences). The auxin response element was the least abundant (4 occurrences), and was identified in the *ZjCDPK3*, *ZjCDPK10*, *ZjCDPK15*, and *ZjCDPK18* promoter regions, respectively.

We identified three stress-related *cis*-acting elements, namely, MBS, TC rich repeats, and LTR. Among them, MBS was observed the most (9 times), followed by LTR (8 times) and TC rich repeats (5 times).

Developmental response *cis*-acting elements, including circadian, CAT box, and ARE, and photoreactive elements such as G-BOX, MRE, and GT1 motifs, were also identified. All *ZjCDPK* promoters contained ARE, except for *ZjCDPK9* and *ZjCDPK18*. *ZjCDPK18* promoter contained a relatively large number of *cis*-acting elements, suggesting that it may participate in more biological and stress-related processes than other *ZjCDPK* members.

### 3.6 Expression profiles of the *ZjCDPKs* during fruit growth

**Figure 6:**
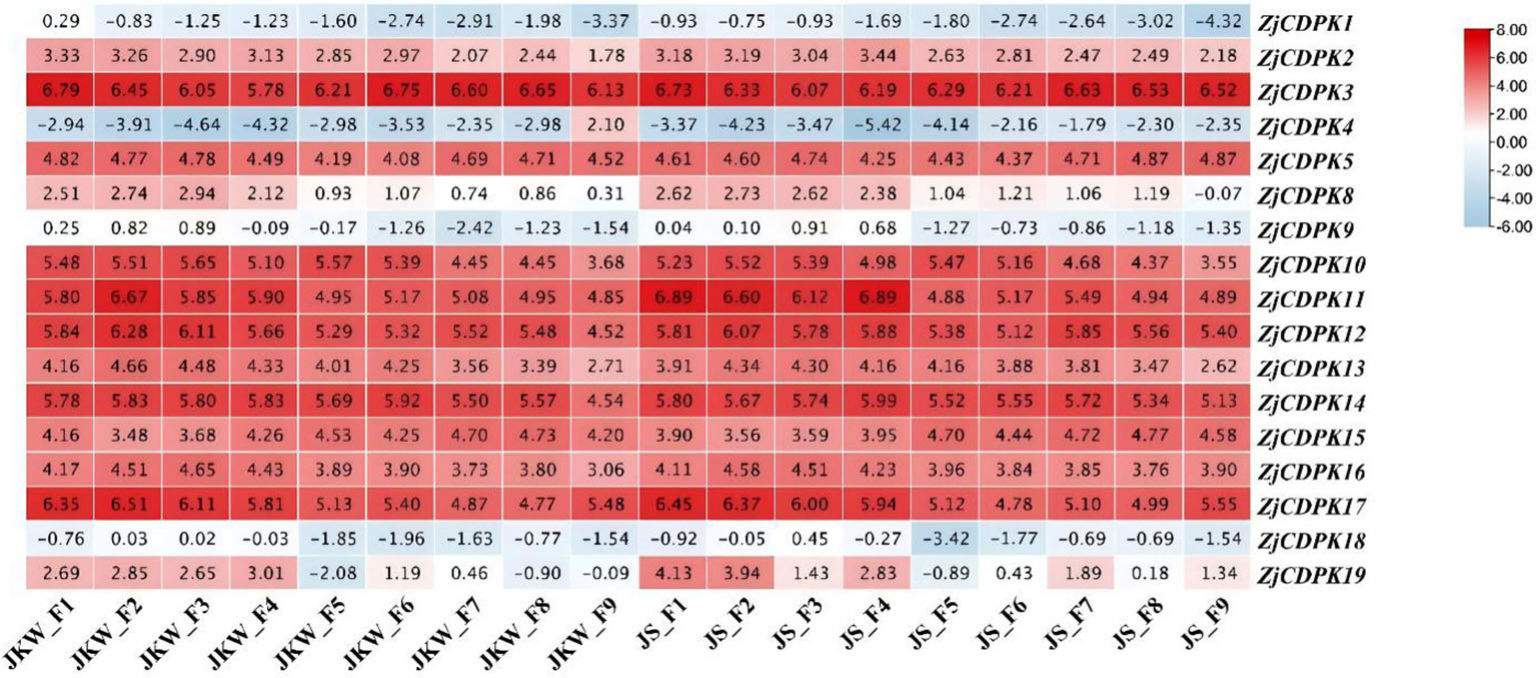
Expression profiles of *ZjCDPKs* during the fruit growth of ‘Jinkuiwang’ (JWK) and ‘Jinsixiaozao’ (JS). F1 to F9 are defined in Section 2.6.

Plant *CDPKs* play important roles in plant growth and development. Thus, to understand how *ZjCDPKs* are involved in fruit growth development, the expression levels of the *ZjCDPKs* in fruit nine growth stages (F1–F9) of ‘Jinkuiwang’ (large fruit size) and ‘Jinsixiaozao’ (small fruit size) were analyzed. The different expression patterns of 17 *ZjCDPKs* were identified (**Figure 6**). The majority of *ZjCDPKs* (e.g., *ZjCDPK1/2/4/18*) showed a down-regulation pattern from F1 to F9 in both cultivars, while other *ZjCDPKs* (e.g., *ZjCDPK 3/5/14/15*) did not exhibit any significant expressing patterns and had high expression levels. Most of the *ZjCDPKs* showed higher expression levels from F1 to F4 and were subsequently down-regulated in the other stages. The expression levels of *ZjCDPK11/14/17/19* increased from F1 to F2 in ‘Jinkuiwang’, but decreased in ‘Jinsixiaozao’. This may indicate the important functions of these *ZjCDPKs* in the different fruit size formation between ‘Jinkkuiwang’ and ‘Jinsixiaozao’.

### 3.7 Expression profiles of *ZjCDPKs* in response to phytoplasma infection

**Figure 7:**
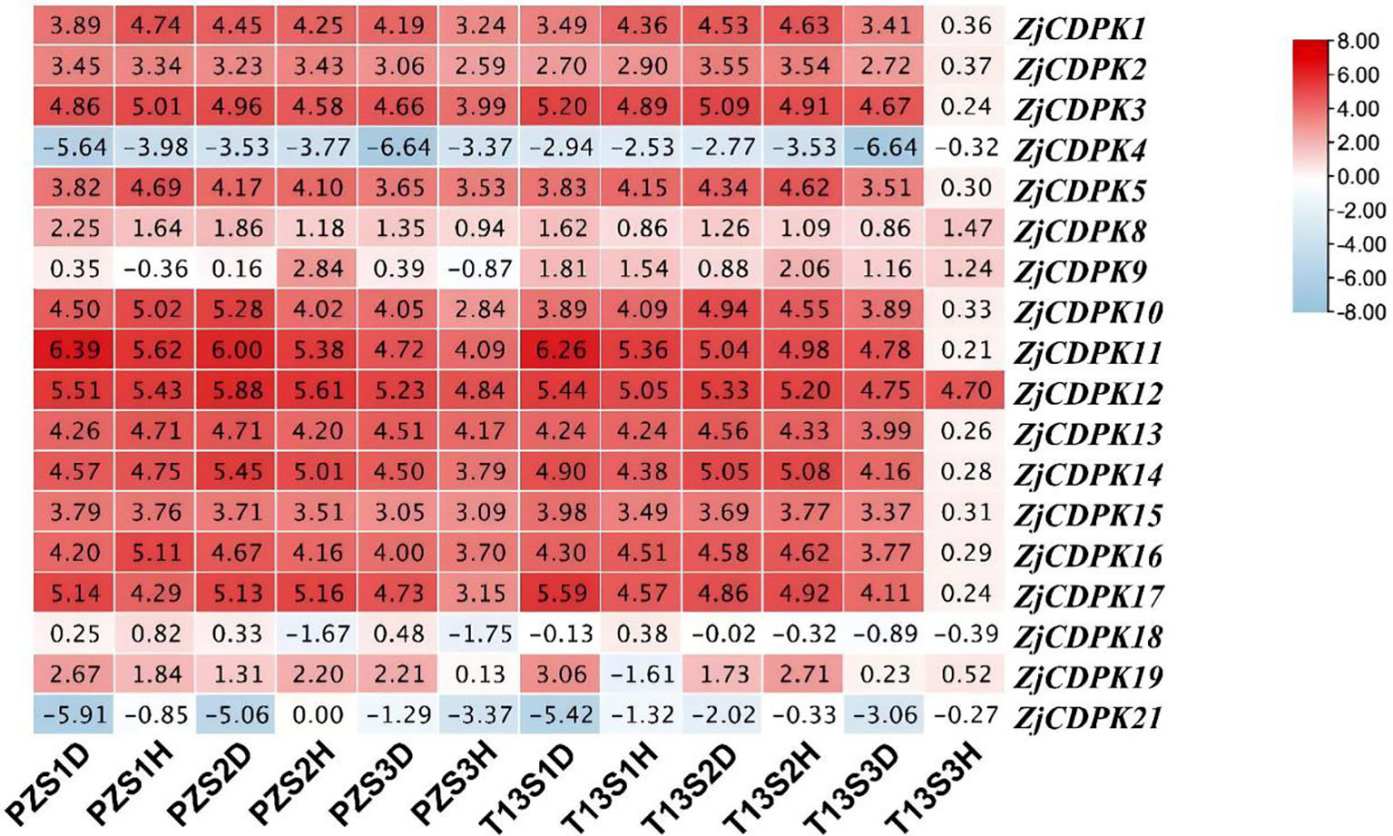
Expression profiles of *ZjCDPKs* in response to phytoplasma infection in ‘Pozao’ (PZ) and ‘T13’ from the S1 to S3 growth stages. H, healthy; D, diseased.

*CDPKs* are involved in the regulating mechanism of biotic stress. Thus, we analyzed the expression profiles of the *ZjCDPKs* in response to phytoplasma infection on the susceptible cultivar ‘Pozao’ and resistant cultivar ‘T13’. As shown in **Figure 7**, compared to the healthy control at stage S1, most of the *ZjCDPKs* exhibited down-regulation in PZ and only *ZjCDPK8/9/11/17/19* was upregulated, while *ZjCDPK3/8/9/11/12/14/15/17/19* showed an upregulating pattern in ‘T13’. As phytoplasma infection growth progressed (S2 to S3) in ‘PZ’, more *ZjCDPKs* were upregulated (e.g., *ZjCDPK1/2/3/5/8/9/10/11/12/13/14/16/17/19/21*) compared to the healthy control. Moreover, most of the *ZjCDPK* expression levels in ‘T13’ did not exhibit any significant changes at S2 and were significantly down-regulated in S3. These results may demonstrate the varying expression patterns of the *ZjCDPKs* involved in the different resistant levels to phytoplasma infection between ‘Pozao’ and ‘T13’.

### 3.8 Expression profiles of *ZjCDPKs* in response to salt stress

**Figure 8:**
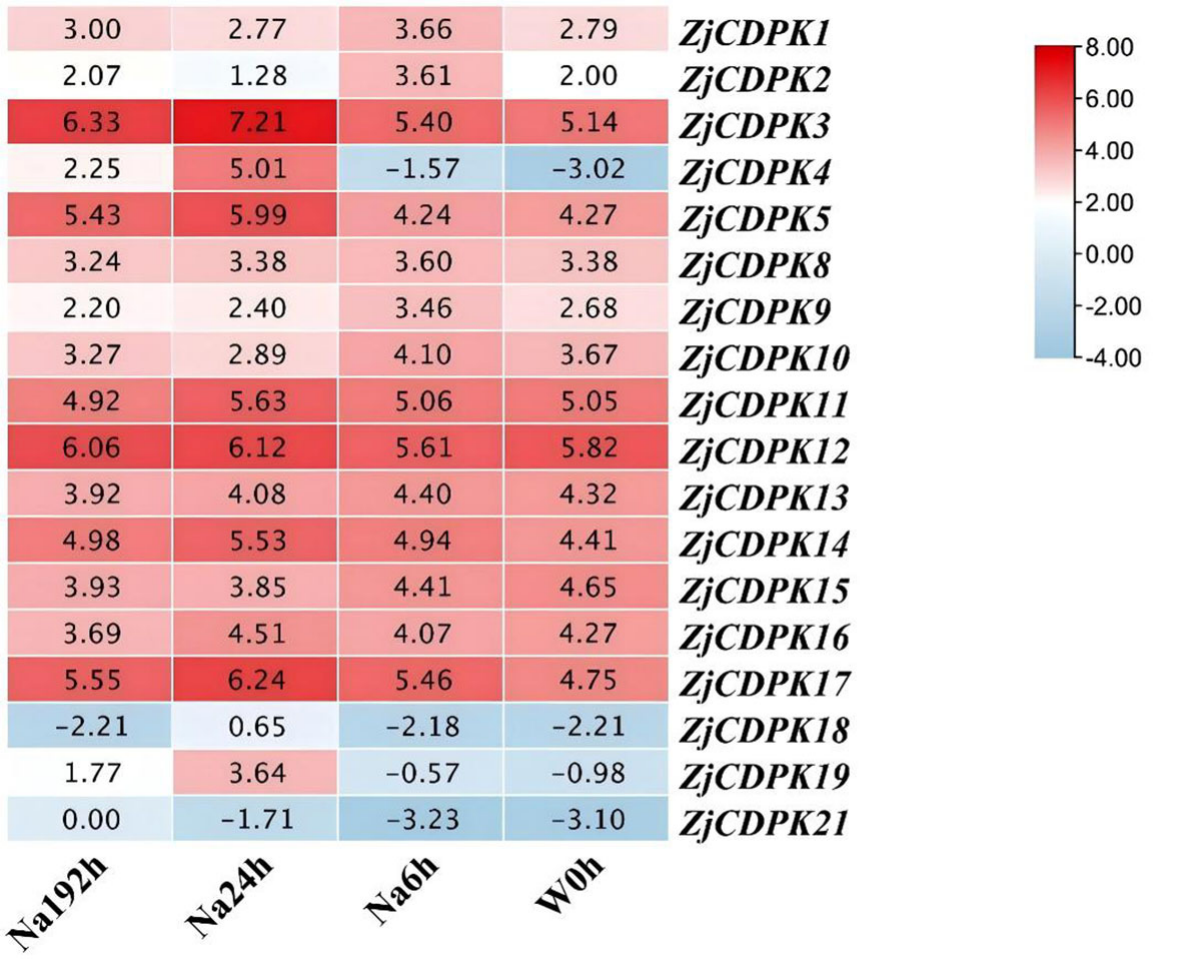
Expression profiles of *ZjCDPKs* in response to salt stress.

*CDPKs* also play important roles in response to abiotic stress. Thus, the expression levels of *ZjCDPKs* in response to salt stress were investigated. In response to salt stress, most of the *ZjCDPKs* were upregulated from 6 h to 24 h, while others initially exhibited a down-regulation pattern and were then regulated at 192 h (**Figure 8**). Among them, *ZjCDPK15* maintained a down-regulation trend from 0 h to 192 h, while the expression levels of *ZjCDPK3/5/11/12/17* increased from 0 h to 192 h and stayed high at 192 h, indicating that these *ZjCDPKs* are positively involved in salt stress.

### 3.9 Expression profiles of *ZjCDPKs* in response to cold stress

**Figure 9:**
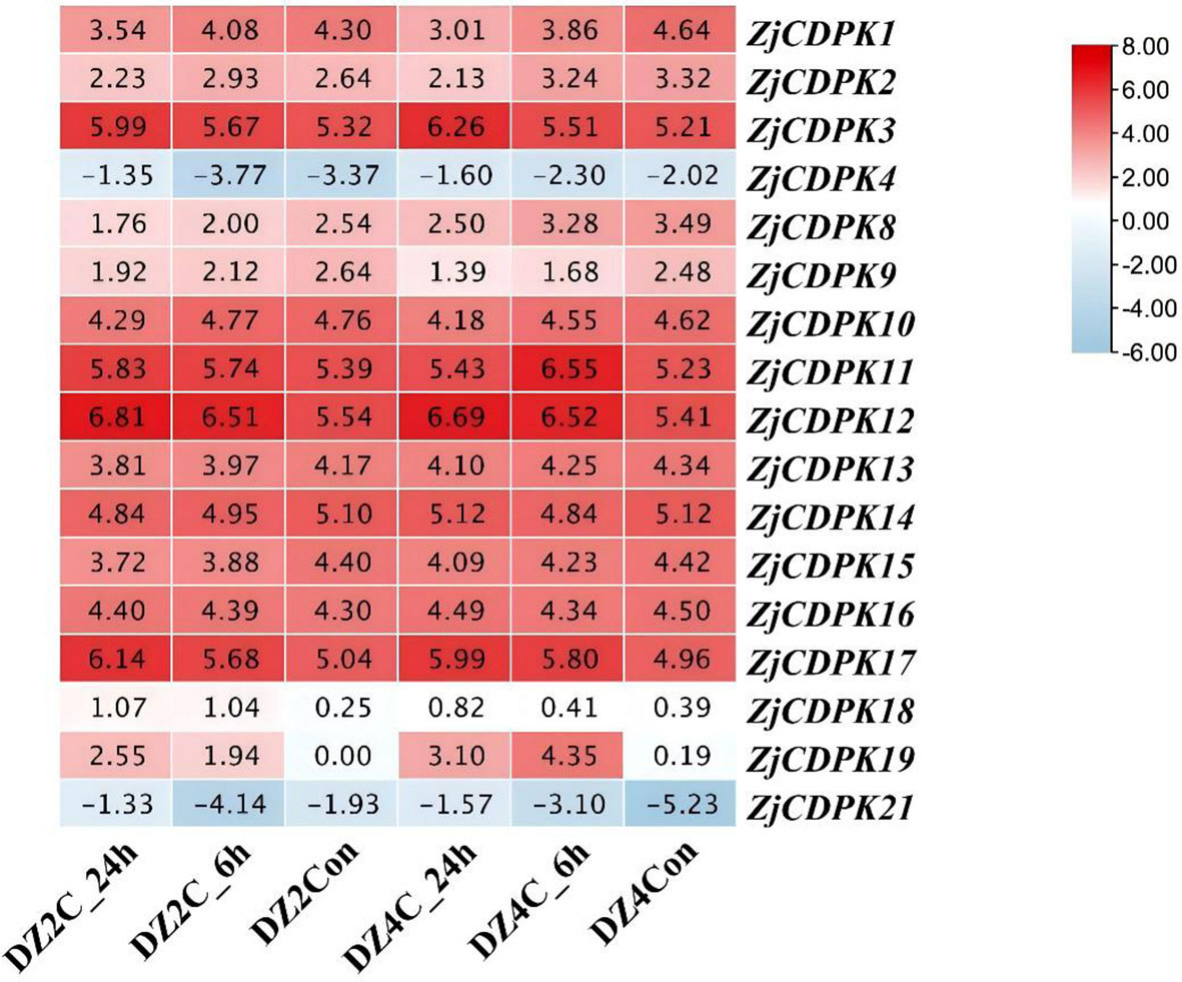
Expression profiles of *ZjCDPKs* in response to cold stress for ‘Dongzao’ and its autotetraploid at 0, 6, and 24 h respectively.

*ZjCDPKs* may have important functions in salt stress, and thus the expression profiles of *ZjCDPKs* in response to cold stress were further analyzed. As shown in **Figure 9**, the expression pattern of *ZjCDPKs* exhibited down- and upregulation patterns in ‘Dongzao’ and its autotetraploid. Among them, most of the *ZjCDPKs*, (e.g., *ZjCDPK1/8/9/10/13/14/15/16*) exhibited a down-regulation trend, while the expression levels of *ZjCDPK3/11/12/17* were upregulated from 0 h to 24 h. In addition, the expression level of *ZjCDPK19* was significantly induced in the ‘Dongzao’ autotetraploid compared to ‘Dongzao’, while the expression level of *ZjCDPK16* was significantly upregulated in ‘Dongzao’ and down-regulated in its autotetraploid. This may indicate the important gene functions of this gene in cold differential resistance between ‘Dongzao’ and its autotetraploid.

### 3.10 Prediction of *ZjCDPK*-related TFs and their expression analysis in response to cold and salt stress

**Figure 10:**
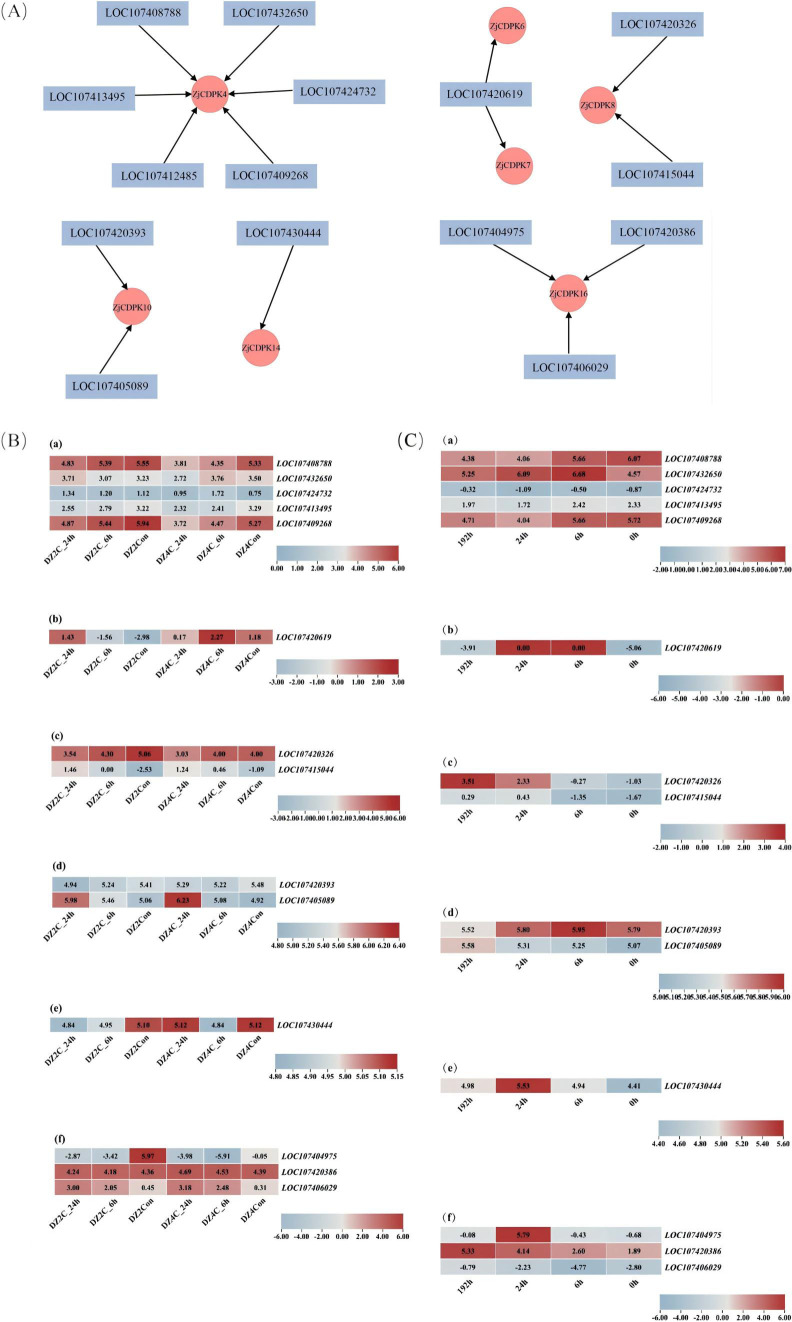
Prediction analysis of *ZjCDPK*-related transcription factors **(A)** and their expression patterns in response to cold **(B)** and salt stress **(C)**.

To explore the regulatory network of *ZjCDPKs* under cold or salt stress, the TFs that could modulate the expression pattern of *ZjCDPKs* were predicted. As shown in **Figure 10A**, *ZjCDPK4/6/7/8/10/14/16* interacted with six, one, one, two, two, one, and three TFs, respectively. The TFs mainly belonged to the ERF, DOF, MADS, and BBM families. Moreover, the expression profiles of these TFs in response to cold and salt stress (**Figures 10B, C**) were analyzed. In response to cold stress, the expression patterns of most TFs showed a down-regulation pattern. However, TFs such as LOC107405089 of *ZjCDPK10* and LOC101706029 of *ZjCDPK16* were significantly upregulated, indicating that these two TFs may play important regulatory roles in cold stress. In addition, under salt stress, the expression patterns of most related TFs showed opposite expressing patterns to that under cold stress. Most TFs were upregulated, while LOC107408788 and LOC107409268 of *ZjCDPK4* were significantly inhibited under salt stress.

## 4 Discussion

CDPKs, as serine/threonine type protein kinases widely found in plants, which function as calcium sensor and responder. Plant *CDPKs* and their mediated signaling cascades regulate plant growth and development, participate in hormone signal transduction, and play a role in abiotic stress response. The *CDPKs* family has undergone a long evolutionary process, which can be traced back to the earliest terrestrial plants, such as pteridophytes and bryophytes (Dekomah et al., 2022a). In the current study, 21 *CDPKs* were identified in the jujube genome using bioinformatics, which is fewer than the number identified in other species such as *Arabidopsis*, poplar, banana, and tomato. The number of gene families may be related to the extensive genomic diversity and tandem and segment duplications that took place in the history of plant evolution (Shi and Zhu, 2022). Thus, the smaller number of *ZjCDPKs* may be attributed to the small number of segment and tandem gene duplications that occurred in the jujube genome.

*CDPK* has four typical conserved domains, and the conserved kinase domain is a typical feature of Ser/Thr protein kinase. Calcium sensors mainly rely on the binding of Ca^2+^ to the EF-hand motif, which is a unique and conserved helical-ring-helical structure (Dekomah et al., 2022a). Our study showed that ZjCDPKs contain two highly conserved domains such as kinase and EF-hand domains. Among them, *ZjCDPKs* have 2–4 conserved EF-hand motifs, which is consistent with those in *Arabidopsis* (Yip Delormel and Boudsocq, 2019). In addition, gene structure analysis showed that the *ZjCDPKs* have multiple introns. The existence of more introns may increase the functional diversity of *ZjCDPKs* through alternative splicing and exon shuffling (Zhu et al., 2016). Moreover, *cis*-acting element analysis revealed that there was numerous hormone, developmental, and stress response elements in the promoters of *ZjCDPKs*. We also found that the methyl jasmonate hormone response element appeared frequently in the *ZjCDPKs*. Methyl jasmonate has many physiological functions, and *CDPKs* that were associated with plant hormones were also involved in the plant defense and development process. For example, *AtCDPK32* can bind and phosphorylate the ABA response transcription factor ABF4, while overexpressing *AtCDPK32* exhibits ABA sensitivity phenotype (Choi et al., 2005). Furthermore, exogenous ABA treatment increases the expression of *BrrCDPK38/42* and *FaCDPK4/11* in brassica and strawberry, respectively (Wang et al., 2017; Crizel et al., 2020). However, studies on the relationship between methyl jasmonate and *CDPKs* are limited, and methyl jasmonate may play an important role in the *CDPK*-mediated biological process of plant development and defense against various stresses.

Although calcium functions are important in plant growth and development, the function of calcium sensors such as *CDPKs* during fruit development has rarely been reported. In our study, we found that most of the *ZjCDPKs* in ‘Jinkuiwang’ and ‘Jinsixiaozao’ showed higher expressing levels during stages F1 to F4 and were then down-regulated during the subsequent stages. Among them, the expression levels of *ZjCDPK11/14/17/19* increased from F1 to F2 in ‘Jinkuiwang’, but decreased in ‘Jinsixiaozao’. ‘Jinkuiwang’ and ‘Jinsixiaozao’ are big- and small-sized fruit cultivars of jujube, respectively. During fruit development, stages F1 to F3 belong to the rapid growth period, and stages F4 to F9 belong to the slow and pre-mature growth period (Zhao et al., 2022). Thus, the different expression levels of *ZjCDPK11/14/17/19* between ‘Jinkuiwang’ and ‘Jinsixiaozao’ at F1 to F2 may determine the fruit size differences. However, further functional verification experiments should be conducted to confirm this hypothesis.

Calcium signaling plays a key role in response to biotic and abiotic stresses. For example, Ca^2+^-permeable channels can be regulated by phytoplasma infection, which can further affect Ca^2+^ signaling in sieve elements (Musetti et al., 2013). In our study, we found that more *ZjCDPKs* were induced with the severe jujube witches’ broom (‘Zaofeng’) disease symptoms in ‘Pozao’ at S3 after phytoplasma infection (Wang et al., 2022). In contrast, when the witches’ broom symptoms recovered in ‘T13’ at S2, the expression levels of most *ZjCDPKs* were maintained constant compared with healthy plants. These results may indicate that the early calcium signal compared with the most expression of *ZjCDPKs* conferred the phytoplasma resistance in ‘T13’. Moreover, *CDPKs* play an important role in cold stress response. For example, *PbCDPK2*, *PbCDPK7*, *PbCDPK10*, and *PbCDPK13* have been associated with post-harvest low-temperature stress in peach. In particular, *PbCDPK7* can interact with PbRBOH4 on the cell membrane, which can induce Ca^2+^-ROS signaling and maintain intracellular ROS homeostasis to reduce chilling injury in peach (Zhao et al., 2022). In rice, *OsCDPK13* (Komatsu et al., 2007), *OsCDPK17* (Almadanim et al., 2017), and *OsCDPK24* (Liu et al., 2018) participate in cold stress responses, while *AtCDPK28* in Arabidopsis (Ding et al., 2022) functions as a positive regulatory factor for cold resistance. In our study, we found that the expression level of *ZjCDPK16* was significantly upregulated in ‘Dongzao’ and down-regulated in its autotetraploid. Previous research reported that ‘Dongzao’ is more cold-tolerant than its autotetraploid (Gao et al., 2021). Therefore, *ZjCDPK16* may have an important function in cold differential resistance between ‘Dongzao’ and its autotetraploid. Moreover, *CDPKs* play a role in salt stress resistance. In *Arabidopsis*, the overexpression of *AtCDPK6* can increase the accumulation of proline and reduce the content of MDA, thereby improving salt stress resistance (Xu et al., 2010). In rice, *OsCDPK7* and *OsCDPK12* play important roles in response to salt stress (Saijo et al., 2001; Asano et al., 2012). *VpCDPK9* is involved in salt stress regulation in grapevine (Zhang et al., 2015). Here, we found that the expression level of *ZjCDPK3/5/11/12/17* increased from 0 h to 192 h and maintained a high level at 192 h in response to salt stress. The transcription factor analysis revealed that most TFs belonging to the MADS and ERF families were significantly upregulated in response to salt stress, demonstrating that TFs could regulate related *ZjCDPKs* to facilitate salt tolerance in sour jujube. The systematic analysis of *ZjCDPKs* can provide important information for further functional analysis of the *CDPKs* in jujube during fruit development and in response to biotic and abiotic stresses. However, the relationship between TFs and *ZjCDPKs*, and their biological functions should be solidified by molecular experiments in the future.

## 5 Conclusion

In this study, a total of 21 *ZjCDPKs* were identified, which are located on eight chromosomes. Gene structure and conserved motif analysis showed that all *ZjCDPKs* have similar gene structures and conserved motifs, except for *ZjCDPK9* and *ZjCDPK21*. All the *CDPKs* from the *Arabidopsis*, rice, tomato, alfalfa, and jujube were divided into eight subgroups and the members of the *ZjCDPKs* were unevenly distributed across these subgroups. Colinear analysis showed that 12 homozygous *CDPKs* were detected between jujube and *Arabidopsis*, and 14 pairs were found between jujube and tomato. Additionally, four types of *cis*-acting elements were identified in the promoters of *ZjCDPKs*, including hormone, stress, development and light response elements were identified. The expression profiles of *ZjCDPKs* in response to phytoplasma infection and cold and salt stresses, during different fruit growth stages indicated that most *ZjCDPKs* were up or down-regulated. Finally, a varying number of TFs could interact with the promoter regions of *ZjCDPK4/6/7/8/10/14/16* and exhibited opposite expression patterns in response to cold and salt stress.

## Data Availability

Publicly available datasets were analyzed in this study. This data can be found here: [https://www.ncbi.nlm.nih.gov/, accession number PRJNA798569].

## Supplementary material

Supplementary Data Sheet 1 Amino acid sequences of *ZjCDPKs*

Supplementary Data Sheet 2 10 motifs were identified from *ZjCDPKs*

Supplementary Data Sheet 3 The *cis*-acting elements identification of *ZjCIPKs*
